# LPA receptor 1 mediates LPA-induced ovarian cancer metastasis: an in vitro and in vivo study

**DOI:** 10.1186/s12885-016-2865-1

**Published:** 2016-11-04

**Authors:** Xuechen Yu, Yuanzhen Zhang, Huijun Chen

**Affiliations:** Department of Gynaecology and Obstetrics, Zhongnan Hospital of Wuhan University, Wuhan, 430071 Hubei China

**Keywords:** Lysophosphatidic acid receptor, Epithelial ovarian cancer, Metastasis

## Abstract

**Background:**

The facts that LPA is present at high concentration in ovarian cancer patients’ ascites and it may serve as a stimulator to cell migration, implicate the role of LPA in the ovarian cancer metastasis. Since LPA mediates various biological functions through its interaction with LPA receptors, we aim to investigate the correlation between the expression of LPA receptors and the metastasis of ovarian cancer.

**Methods:**

To test whether the LPA responsiveness correlated with the metastatic capability of ovarian cancer cells, we performed LPA induced invasion assay and peritoneal metastatic colonization assay with a panel of established human ovarian cancer cell lines. The expression of LPAR1-3 in different ovarian cancer lines was examined by qRT-PCR. We also tested the effects of LPAR1 inhibition or overexpression on ovarian cancer cell's invasiveness. To confirm our laboratory results, we detected LPARs expression in specimens from 52 ovarian cancer patients by qRT-PCR and immunohistochemistry.

**Results:**

Thirteen ovarian cancer cells were enrolled in the invasion assay. Ovarian cancer cell lines which responded well to LPA-induced invasion, also displayed good capability for metastatic colonization. On the contrary, cell lines with poor LPA responsiveness showed inferior metastatic potential in peritoneal colonization assay. High expression level of LPAR1 was detected in all of the metastatic ovarian cancer cell lines. *T*-test showed that LPAR1, not LPAR2 or LPAR3, expression was significantly higher in the metastatic cell lines than in the non-metastatic cell lines (*P* = 0.003). Furthermore, silencing LPAR1 alone could significantly reduce LPA-induced invasion (*P* < 0.001). Finally, we analyzed the correlation between the LPARs expression and clinicopathological features of the clinical cases. It indicated that LPAR1 expression rate increased significantly along with the more advanced stages (stage I: 16.67 %; II 50.00 %; III: 75.00 %; and IV: 100.00 %; *P* = 0.003). Besides that, LPAR1 expression was detected in all the 13 cases with abdominal metastasis more than 2 cm, 10 cases with retroperitoneal lymph node metastasis and 6 cases with hepatic metastasis. Moreover, the expression rate of LPAR2 significantly increased in ovarian cancer than in normal specimens (*P* = 0.039). LPAR3 expression showed the same trend as LPAR2, though the difference is not statistically significant (*P* = 0.275). Besides that LPAR2 and LPAR3 expression increased along with poorer differentiation (*P* = 0.002, *P* = 0.034, respectively).

**Conclusions:**

Metastatic capability of ovarian cancer cells correlated well with their responsiveness to LPA for cell invasion. LPAR1 acts as the main mediator responsible for LPA-stimulated ovarian cancer cell invasion. LPAR2 and LPAR3 might play an role in carcinogenesis of ovarian cancer.

## Background

Ovarian cancer is the most lethal disease among all the gynecological cancers. The high mortality rate of ovarian cancer is mainly due to the complications of metastasis. Once the epithelial cells covering the ovaries undergo neoplastic transformation, they exfoliate from the primary tumor and disseminate to the peritoneal cavity through implantation pattern. It is widely recognized that the accumulation of malignant ascites is one of the most typical behaviors of ovarian cancers and may help the cancer cells to seed the abdominal cavity organs with tumor implants [1].

Lysophosphatic acid (LPA) is a growth factor-like phospholipid that elicits multiple cellular events, including cell migration, proliferation, and survival [2, 3]. LPA is uniquely associated with ovarian malignancies, as signified by its presence at high concentrations in the ascites of ovarian cancer patients [4, 5], and its production and secretion into the peritoneal cavity by ovarian cancer cells [6, 7] as well as mesothelial cells [8]. Moreover, LPA stimulates ovarian cancer cell migration [9, 10], triggers protease production/activation in ovarian cancer cells [11, 12], induces Cox-2 expression [13], and facilitates angiogenesis through the induction of various proangiogenic factors, such as VEGF [14], IL8 [15], and Groα [16]. These findings implicate the role of peritoneal fluid- or ascites-borne LPA as a potent promoter of peritoneal metastasis of ovarian cancer. The cellular responses of LPA are mediated by a group of G protein-coupled receptors (GPCRs), in which LPAR1, LPAR2, and LPAR3 are best characterized and widely expressed [17, 18]. Previous reports have suggested that an upregulated expression of LPAR may be involved in the mechanism underlying tumor growth and metastasis [19, 20]. However, a few studies have focused on the correlation between LPA receptors and ovarian cancers.

It has been long recognized that the ability of cancer cells to invade the surrounding tissues is essential for metastasis. The facts that the levels of LPA are elevated in the ascites in ovarian cancer patients, and that LPA may serve as a stimulator to cell migration as well as protease production/activation, prompted us to hypothesize that LPA-stimulated cancer cell invasion may play a critical role in ovarian cancer metastasis. Here, we presented that the peritoneal metastatic colonization of ovarian cancer cells is associated with their ability to respond to LPA for cell invasion. Besides, we demonstrated that LPAR1 acts as the main mediator responsible for LPA-stimulated ovarian cancer cell invasion. LPAR2 and LPAR3 might play an role in carcinogenesis of ovarian cancer.

## Methods

### Cells and antibodies

The human ovarian cancer cell lines, ES2, OVCAR429, HEY, OVCAR433, OVCAR5, SK-OV3, OCC1, OVCAR3, TOV21G, HEC1A, IGROV1, A2780, and OVCAR4 were provided by the Department of Biochemistry and Molecular Biology, Georgia Regents University (Georgia, USA) as a kind gift. Cells were cultured in Dulbecco's modified Eagle’s medium (DMEM) supplied with 10 % (w/v) fetal bovine serum (FBS) at 37 °C in a humidified incubator containing 5 % CO_2_. LPA was purchased from Avantis Lipid (Alabaster, AL). DMEM, serum, and other cell culture supplies were purchased from Maixin Biotechnology (Fuzhou, China).

### Clinical specimens

Clinical specimens were obtained from 52 primary epithelial ovarian cancer patients and 15 non-tumor patients, who underwent ovariectomy due to other diseases at the Department of Obstetrics and Gynecology, Zhongnan Hospital of Wuhan University, Wuhan, China, from December, 2011 to December, 2015. All experiments were approved by the Ethics Committee of Wuhan University. The fresh specimens were frozen in liquid nitrogen and stored at −80 °C. The formalin-fixed/paraffin-embedded samples were also collected. Surgical pathological stages were assessed according to the International Federation of Gynecology and Obstetrics (FIGO) criteria. The range of carcinoma invasion and metastasis were confirmed by surgical exploration and postoperative pathological examination.

### Matrigel invasion assay

The effect of LPA on cell invasion ability was measured by Matrigel invasion assay (Corning Incorporated, MA, USA). LPA dissolved in serum-free medium (20 μM) was added into the underwells of invasion plates as a chemoattractant to induce cell invasion, while the serum-free medium without LPA was used as control. Serum-starved ovarian cancer cells (10^5^/well, in log phase) were detached and plated into the upper Matrigel-coated invasion chambers. The cells were then allowed to invade for 48 h. The remaining cells in the chambers were removed by cotton swabs and the invaded cells on the lower surface of the chambers were fixed and stained with crystal violet. Subsequently, the crystal violet-stained cells were solubilized with 10 % acetic acid and quantitated on a microplate reader at 600 nm. Fold increase in cell invasion was calculated to evaluate the effects of LPAR1-3 silence on cells’ responsiveness for LPA(OD_600_ LPA-induced cell invasion /OD_600_ base cell invasion).

### Peritoneal metastatic colonization assay

Six-week-old athymic female homozygous nu/nu mice were purchased from and cultured in the Animal Biosafety Level-3 Laboratory of Wuhan University under sterile environment. Ovarian cancer cells in log-phase were trypsinized, washed, and resuspended in PBS. The mice were intraperitoneally injected with different cell lines (10^7^ cells/mice), and monitored for five weeks. The mice were then sacrificed and autopsied. Visible metastatic implants were observed and photographed. All animal experiment procedures were approved by the Animal Center of Wuhan University.

### RNA inference and overexpression

To test the role of LPARs in LPA-induced cell invasion, specific shRNAs to LPAR1_−3_ were designed and introduced into HEY and SK-OV3 cells. The shRNAs for each target genes were designed with the aid of web-based Invitrogen Block-It program and inserted into pLV-shRNA vector (Biosettia). The target sequences were as follows: sh-LPAR1: 5’-GGATACCATGATGAGTCTTCT-3’, sh-LPAR2: 5’-GCCTGGTCAAGACTGTTGTCA -3’, sh-LPAR3: 5’-GCCAAGGTGCAGTCTGCAATA-3’. Matrigel invasion assay was performed in LPAR1-3 knockdown cell lines, respectively. Vector containing coding sequence of LPAR1 were got from Biochemistry and Molecular Biology Department, Georgia Regents University, as a gift. Lentiviral vector encoding LPAR1 were prepared by subcloning the coding sequence into pCDH-CMV-MCS-EF1-Puro vector. TOV21G and OVCAR3 cells were respectively infected with lentiviral vectors encoding LPAR1 for 2 days and chosed by puromycin. The efficiency of LPAR1 over-expression was tested by RT-PCT. After 2 days starvation, cells were stimulated with 20 μM LPA and followed by the Matrigel invasion assay.

### RNA isolation and qRT-PCR analysis

Total RNA was extracted with Trizol (Invitrogen, Carlsbad, CA, USA) and treated by DNase I to remove the remaining genomic DNA. The concentration of RNA was determined at 260 nm and 280 nm by spectrophotometry, while the purity was detected by denaturing agarose gel electrophoresis. DNase I-treated RNA (2 μg) was reverse transcribed with SuperScriptase II. Generated cDNA was subjected to real-time PCR to measure LPAR1, LPAR2, LPAR3, and GAPDH levels with the respective TaqMan probes and TaqMan^@^Universal PCR Master Mix Kits (Applied Biosystems, Foster City, CA, USA). The reaction was performed using ABI 9500Fast Real-time machine and the conditions were as follows: 95 °C for 10 min, followed by 40 cycles at 95 °C for 15 s and 60 °C for 1 min. The expression levels of the target gene were standardized by comparing the Ct value of target genes to the GAPDH, and presented as 2 ^[Ct(GAPDH) - Ct(target gene)]^ [21].

### Immunohistochemistry

Paraffin-embedded sections (4 μm-thick) were deparaffinized and rehydrated. Hydrogen peroxide treatment was used to block endogenous peroxidase activity. Sections were blocked with goat serum and incubated with polyclonal antibodies against LPAR1(Cat#: PAB10126), LPAR2(Cat#: A-ALS10695) or LPAR3 (Cat#: A-ALS10242). Primary antibodies were purchased from Amyjet Scientific Inc. Antigens were visualized by streptavidin-biotin-peroxidase complex method. Immunostaining was evaluated by two pathologists without knowledge of patients’ clinical information. All three antigens were found to be localized in cytoplasm. Extent of immunostaining was graded based on the percentage of cells displaying staining. “-” is considered as negative staining (<10); “+”, “++” and “+++” were considered as positive staining (10-25 %, 25-50 % and >50 % respectively).

### Statistical analysis

Statistical analyses of the invasion assay were performed by ANOVA and independent *t* test. Chi-square test and Fisher's exact test were used to compare covariates between LPARs expression and clinicopathological parameters. All of the statistical tests were two-sided and *P*-values of less than 0.05 were indicated as statistically significant.

## Results

### Effects of LPA on the migration of ovarian cancer cell lines

**Fig. 1:**
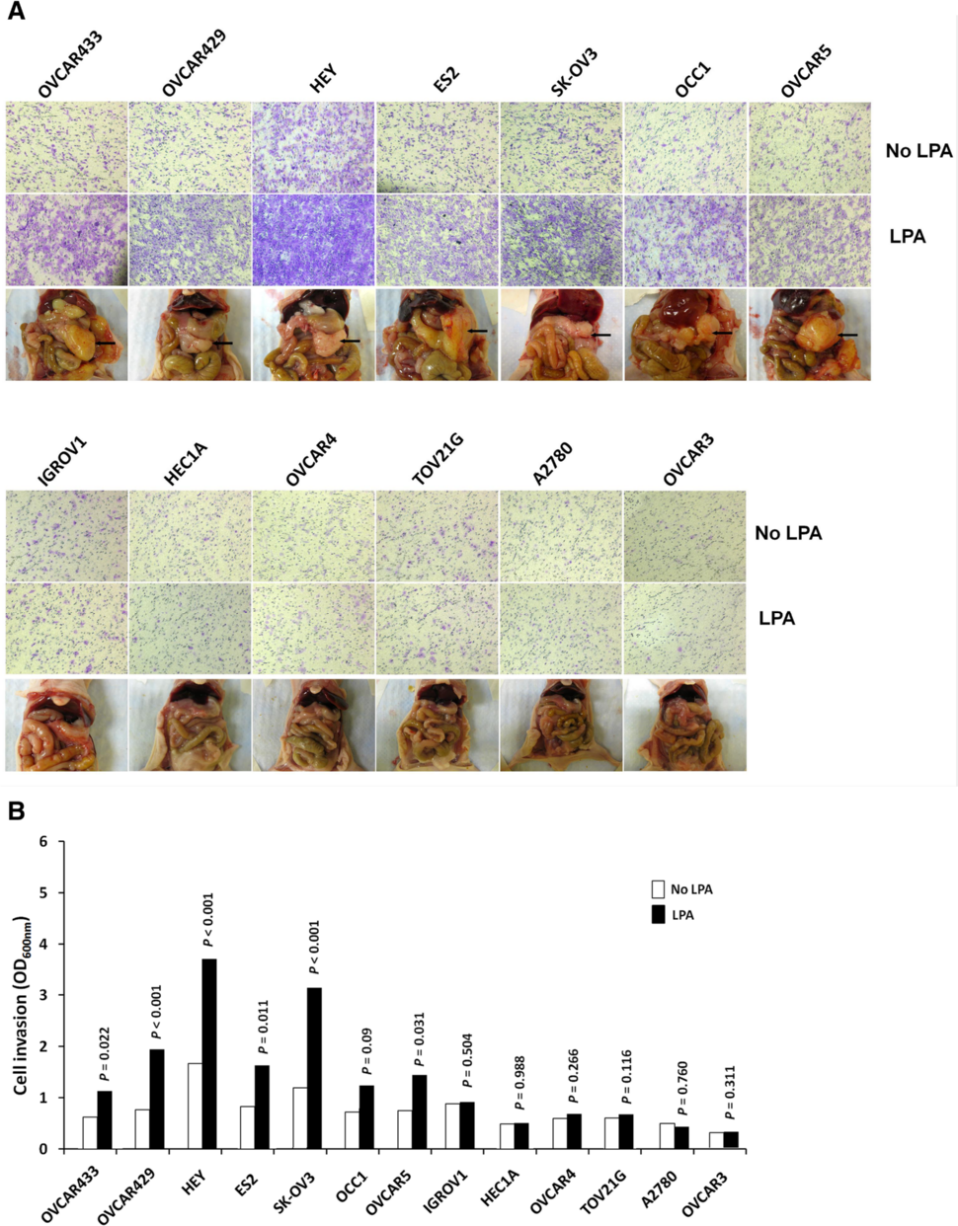
Correlation between response to LPA-induced invasion and metastatic colonization potential of ovarian cancer cells. **a** Invasion of ovarian cancer cells stimulated by LPA. Cell invasion was measured using Matrigel invasion assay with/without 20 μM LPA in the underwells. Peritoneal metastatic colonization assay. The nu/nu mice were intraperitoneally injected with different cell lines (107cells/mice), and autopsied five weeks later. Visible metastatic implants were observed and photographed. **b** The invaded cells were stained with crystal violet, dissolved in 10 % acetic acid and quantitated with a microplate reader at 600 nm. All samples were performed in triplicate. Data are expressed as the means ± SE

To test whether the LPA response correlated with the metastatic capability of ovarian cancer cells, we performed LPA-induced invasion assay and peritoneal metastatic colonization assay with a panel of established human ovarian cancer cell lines. Invasion assay revealed that not all the cell lines responded well to LPA. A significant increase in cell invasion of ES2, OVCAR429, HEY, OVCAR433, OVCAR5, SK-OV3, and OCC1 lines was observed with LPA stimulation. However, the OVCAR3, TOV21G, HEC1A, IGROV1, A2780, and OVCAR4 cell lines showed poor or no response to LPA with regard to cell invasion. Subsequently, the metastatic potential of these cell lines was assessed by analyzing metastatic colonization with a well established peritoneal seeding model [22, 23]. Animals injected with ES2, OVCAR429, HEY, OVCAR433, OVCAR5, SK-OV3, and OCC1 lines (capable of responding to LPA for cell migration) displayed overt metastatic implants on omentum, liver, and diaphragm, which resemble human ovarian cancer; and these lines were designated as metastatic. In contrast, metastatic colonization was not observed in animals receiving OVCAR3, TOV21G, HEC1A, IGROV1, A2780, and OVCAR4 (incapable of responding to LPA for cell migration), and these lines were referred to as non-metastatic (Fig. 1). These results, therefore, demonstrated that the LPA response of ovarian cancer cells correlated well with their metastatic potentials.

### LPAR mRNA expression in ovarian cancer cell lines

LPA-induced cellular events can be potentially mediated by multiple LPA receptor subtypes [17, 18]. In the present study, we focused on LPAR1-3 for their possible role in ovarian cancer cell invasion and metastasis, as these are the most characterized lines and their aberrant expression have been detected in various cancer tissues. The expressions of LPAR1-3 in different ovarian cancer lines were examined by qRT-PCR. As shown in Table 1, high expression levels of LPAR1 were revealed in all the metastatic ovarian cancer cells (ES2, OVCAR429, HEY, OVCAR433, OVCAR5, SK-OV3, and OCC1). Furthermore, the *t*-test established that LPAR1 expression was significantly higher in metastatic cell lines than in non-metastatic cell lines (*P* = 0.003). However, we also noticed that not all of the non-metastatic cell lines were low level LPAR1 expressing ones. OVCAR3, IGROV1 and TOV21G also expressed a moderate level of LPAR1. On the contrary, we did not detect any statistically significant difference in LPAR2 and LPAR3 transcript levels between metastatic lines and non-metastatic lines. These results indicate the possibility of LPAR1 as the key factor for ovarian cancer cell metastasis.

**Table 1:** Expression levels of the three LPARs in ovarian cancer cell lines

	LPAR1		LPAR2		LPAR3	
	mRNA expression	*P* value	mRNA expression	*P* value	mRNA expression	*P* value
Invasive ovarian cancer cells						
ES2	1.591 ± 0.033		0.075 ± 0.031		0.702 ± 0.047	
OVCAR429	2.235 ± 0.014		1.602 ± 0.012		0.086 ± 0.071	
HEY	0.770 ± 0.038		0.005 ± 0.063		0.261 ± 0.032	
OVCAR433	1.919 ± 0.013		0.847 ± 0.085		0.064 ± 0.044	
OVCAR5	0.715 ± 0.012		0.140 ± 0.033		0.005 ± 0.017	
SKOV3	0.633 ± 0.078		0.087 ± 0.087		0.177 ± 0.105	
OCC1	1.165 ± 0.014		0.138 ± 0.011		2.751 ± 0.037	
Non-invasive ovarian cancer cells		0.003*		0.246		0.804
OVCAR3	0.252 ± 0.011		0.192 ± 0.021		0.451 ± 0.022	
HEC1A	0.002 ± 0.025		0.053 ± 0.045		0.001 ± 0.065	
IGROV1	0.383 ± 0.045		0.129 ± 0.015		0.082 ± 0.045	
TOV21G	0.274 ± 0.024		0.002 ± 0.024		0.026 ± 0.008	
A2780	0.007 ± 0.014		0.081 ± 0.033		2.070 ± 0.102	
OVCAR4	0.001 ± 0.013		0.268 ± 0.008		0.067 ± 0.073	

* *P* < 0.003 indicates statistically significant difference

### LPAR1 is responsible for LPA-induced ovarian cancer cell invasion

**Fig. 2:**
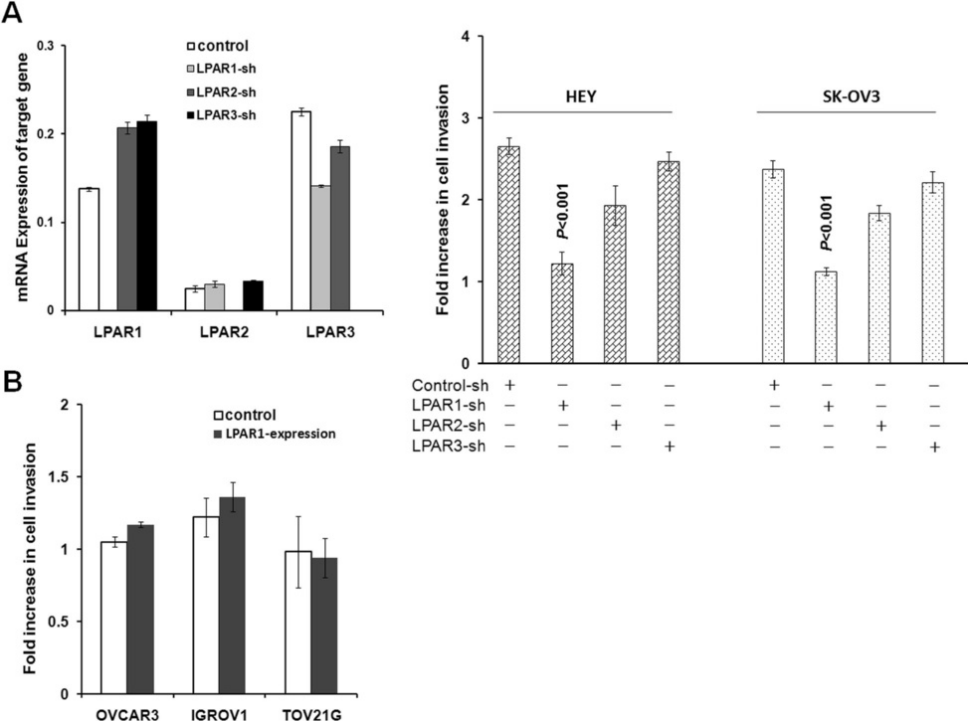
Effects of silencing LPA1-3 on ovarian cancer cells’ response for LPA-induced invasion. **a** SK-OV3 and HEY cells were transduced with control or LPAR1-3 shRNAs and then analyzed for cell invasion with or without 20 μM LPA contained in the underwells. Results are presented as fold increase of cell invasion (OD600 LPA-induced cell invasion /OD600 base cell invasion). Data are means SE. *n* = 3. Differences between groups were assessed using Student *t* test. **b** We lentivirally overexpressed LPAR1 in I IGROV1, TOV21G and OVCAR3 lines. Enforced LPAR1 expression was unable to render non-metastatic IGROV1, TOV21G and OVCAR3 cells responding to LPA for cell invasion

Specific shRNAs targeting LPAR1-3 were designed and introduced into HEY and SK-OV3 cells. The efficiency of target gene knockdown was confirmed by qRT-PCR (Fig. 2). Silencing LPAR1 alone significantly reduced LPA-induced cell invasion (*P* < 0.001). On the contrary, LPAR2-shRNA displayed slight and LPAR3-shRNA exhibited no effect on LPA-induced cell invasion (*P* = 0.193, *P* = 0.248 respectively). These results indicated that LPA-stimulated ovarian cancer cell invasion was mediated mainly through LPAR1. Our data above also showed some cell lines with moderate LPAR1 expression were non-metastatic ovarian cells. To rule out the possibility that LPA non-responsive lines contained potential function-impairing mutation in LPAR1 sequence, we lentivirally transduced LPAR1 into IGROV1, TOV21G and OVCAR3 lines. However, these lines with LPAR1 overexpression remained non-responsive to LPA for cell invasion. These results indicate that the inability of LPA to stimulate cell invasion in LPA non-responsive line is not at the step of LPA receptors.

### LPAR protein expression in clinical specimens

**Fig. 3:**
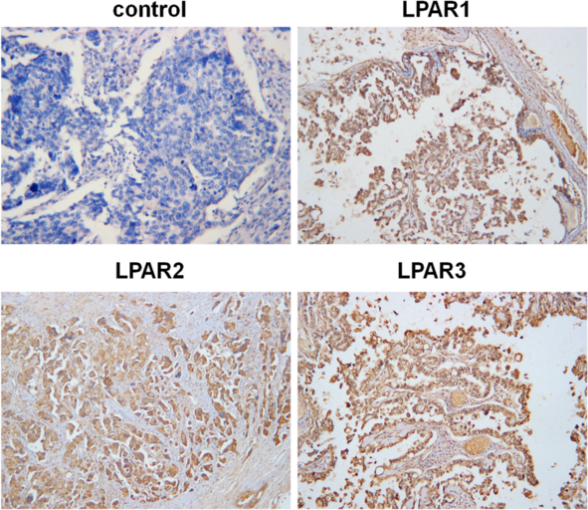
Immunohistochemistry of LPAR, LPAR2 and LPAR3 on ovarian cancer specimens. (×100)

As laboratory studies may not recapitulate clinical ovarian malignancy, we extended our study by detecting LPARs expression in fresh specimens from patients by qRT-PCR and immunohistochemistry (Additional file 1: Table S1). The qRT-PCR results showed that LPAR1, LPAR2, and LPAR3 were positive in 75.00 %, 12.50 %, and 6.25 % in the 15 of the normal ovarian specimens, respectively; and 69.23 %, 42.31 %, 17.31 % in the 52 of the ovarian cancer specimens, respectively. The expression rate of LPAR2 was much higher in ovarian cancer specimens than in normal ones (*P* = 0.039). LPAR3 expression rate is also increased in cancer than in normal specimens, though the difference is not statistically significant (*P* = 0.275). On the contrary, no significant difference in LPAR1 expression between normal or cancer specimens were observed (*P* = 0.658). To further evaluate the role of LPARs in ovarian cancer metastasis, we analyzed the relationships between the expression of LPARs and clinicopathological features. As presented in Table 2, LPAR1 expression rate increased significantly with more advanced clinical stages (stage I: 16.67 %; II 50.00 %; III: 75.00 %; and IV: 100.00 %; *P* = 0.003). Besides, LPAR1 expression was detected in all the 13 cases with abdominal metastasis, more than 2 cm; 16 cases with retroperitoneal lymph node metastasis; and 6 cases with hepatic metastasis. We also found that LPAR2 and LPAR3 expression rate increased along with the more advanced pathologic grades (*P* = 0.002, *P* = 0.034, respectively). The immunohistochemistry also demonstrated that LPAR1 positive percentage increased along with the clinical stages (stage I: 15.38 %; II 37.50 %; III: 66.67 %; and IV: 83.33 %; *P* = 0.002), while LPAR2 and LPAR3 positive percentage increased along with the pathologic grades (*P* = 0.005, *P* = 0.025, respectively) (Fig. 3). These results were in accordance with the data from RT-PCR.

**Table 2:** Relationship between clinical characteristics of ovarian cancer patients and the expression of LPAR1-3

	Total (N)	LPAR1-Positive (%)	*P* value	LPAR2- Positive (%)	*P* value	LPAR3-Positive (%)	*P* value
	52	36 (69.23)		22 (42.31)		9 (17.31)	
Histological subtypes	0		0.664		0.587		0.542
Serous	26	20 (76.925)		11 (42.31)		4 (15.38)	
Mucinous	8	5 (62.5 %)		3 (37.50)		2 (25.00)	
Endometroid	12	7 (58.33 %)		4 (33.33)		3 (25.00)	
Clear cell/Undifferentiated	6	4 (66.67 %)		4 (66.67)		0 (0)	
Differentiation			0.415		0.002^*^		0.034^*^
High	13	10 (76.92)		2 (15.38)		2 (15.38)	
Moderate	20	12 (60.00)		6 (30.00)		2 (10.00)	
Poor	19	14 (73.68)		14 (73.68)		5 (26.32)	
Clinical stage			0.003^*^		0.712		0.678
FIGO I	6	1 (16.67)		3 (50.00)		2 (33.33)	
FIGO II	8	4 (50.00)		3 (37.5)		2 (25.00)	
FIGO III	28	21 (75.00)		12 (42.86)		4 (14.29)	
FIGO IV	10	10 (100.00)		4 (40.00)		1 (10.00)	

* P <0.05 indicates statistically significant difference

## Discussion

LPA is present at high concentrations in the ascites in patients with ovarian cancer. A number of experimental studies have demonstrated that LPA can promote ovarian cancer cell proliferation/survival, and induce the production of proangiogenic factors [14, 15] and proteases [11, 12]. In this study, LPA was shown to be a potent invasion stimulator for various ovarian cancer cell lines. These findings suggested a possibility of the involvement of peritoneal fluid or ascites-contained LPA in spreading and disseminating ovarian cancer cells. As cell invasion is one of the most crucial components of cancer metastasis, we reasoned that the ability of ovarian cancer cells to respond to LPA for cell invasion may be essential for their peritoneal metastasis. This theory was further supported by the observation that LPA-stimulated cell invasion presented an excellent correlation with peritoneal metastatic colonization of ovarian cancer cells.

LPA mediates various biological responses through its interaction with LPA receptors, namely LPAR1, LPAR2, and LPAR3, which belong to the G protein-coupled receptor (GPCR) superfamily. By binding to LPARs, LPA can activate three distinct G-protein subfamilies (G_12/13_, G_i_ and G_q_), and then stimulate multiple downstream signaling pathways including Ras-MAPK, Rho GTPase, and KT/PKB. Eventually, it can trigger a series of biological events [24]. Most of the previous studies about LPA-induced ovarian cancer metastasis have emphasized on the downstream regulatory factors. Rare reports systematically studied the correlation between LPA receptors and ovarian cancer metastasis, and the role of LPARs in cancer metastasis is still under controversial. Park et al. reported an elevation in the expression levels of LPAR1 and matrix metalloproteinase (MMP)-9 due to LPA, which subsequently induced hepatocellular carcinoma (HCC) cell invasion [25]. Mayumi Komachi’ study indicated that LPA1 receptors mediate stimulation, whereas LPA2 receptors mediate inhibition, of migration of pancreatic cancer cells in response to lysophosphatidic acid and malignant ascites [26]. Chen et al. suggested that LPAR2 (EDG4) and LPAR1 (EDG2) could cooperatively promote an efficient Rho-dependent chemotaxis in breast carcinoma cells, while they observed LPA_2_ to be less efficacious [27]. In a similar study, Yu et al. found that the expression of LPA_2_ and LPA_3_ mRNAs were higher in most ovarian cancer cell lines as compared with normal ovarian epithelial cells. However, in our study, we demonstrated that LPAR1 expression in invasive ovarian cancer cells was significantly higher than in non-invasive ones; while the expression of LPAR2 and LPAR3 had no statistical correlation with the metastatic potential of ovarian cancer cells. This difference may be attributed to differences in the cell lineage. Moreover, we observed that LPAR1 was highly expressed in all invasive ovarian cancer cells and all the three low LPAR1 expressing cells are non-invasive ones through there are still three of non-invasive lines expressing a moderate level of LPAR1. Besides that, silencing LPAR1 alone could significantly reduce LPA-induced invasion. Our in vitro data suggested that LPAR1 is the major receptor of LPA-induced ovarian cancer metastasis. As multiple signaling pathways are involved in tumor cell migration, there may also be other key factors in signal transmission of ovarian cancer besides LPAR1. Lack of certain downstream factors may lead to the depression of tumor cell invasion. This may explain why LPAR1 overexpression could not render IGROV1, TOV21G and OVCAR3 lines capable of responding to LPA for invasion in our study.

LPA receptors are widely distributed in embryos, tissues, and cell lines, and each subtype has a distinct specificity. Recent studies have evaluated the expression of LPA receptors in clinical specimens. For instance, Shida et al. observed a reduced expression of LPAR1 and increased expression of LPAR2 in colorectal cancers as compared with normal mucosa. The ratio of LPA_2_/LPA_1_ in cancer tissues contributes to pathogenesis in cancer biology [28]. The expression levels of LPA receptors in human epithelial ovarian neoplasms were detected using RT-PCR by Wang et al., and LPAR2 and LPAR3 were found to be overexpressed in ovarian cancer when compared with tissues from normal ovaries and benign ovarian tumors [29]. In our study, we also found that the expression rate of LPAR2 increased in ovarian cancer than in normal specimens. LPAR3 expression showed the same trend as LPAR2, though the difference is not statistically significant. Besides that LPAR2 and LPAR3 expression increased along with poorer differentiation. These data suggested that LPAR2 and LPAR3 might play an role in carcinogenesis but not in cancer cell invasion. On the contrary, the expression of LPAR1 did not show any difference between cancer and normal tissues; however, it was observed to increase with more advanced clinical stages. The expression of LPAR1 was further revealed in cases with abdominal metastasis (greater than 2 cm), retroperitoneal lymph node metastasis, and hepatic metastasis. This observation corroborated the results generated from the cell lines that LPAR1 is the main receptor responsible for the LPA-induced ovarian cancer metastasis.

## Conclusions

In summary, our study demonstrated that LPA response might be a prerequisite for peritoneal metastasis of ovarian cancer cells, and that LPAR1 is the major mediator for LPA-induced ovarian cancer invasion as well as metastasis. Although our results were supported with established ovarian cancer cell lines, which may not completely simulate the clinical settings, the consistency seen in multiple cell lines, the convergence of loss- and gain-of-function findings, and especially, the significant correlation observed between LPAR1 expression and advanced disease stage/wider spreading range strongly argue against any confounding influence derived from our experimental studies. As LPA receptors are located on the cell surface and easily influenced by drugs, there lies immense potential in developing a therapeutic approach by targeting LPAR1 and its downstream factors.

## Abbreviations

LPA: Lysophosphatic acid
GPCRs: G protein-coupled receptors
DMEM: Dulbecco’s modified Eagle’s medium
FBSL: Fetal bovine serum
FIGO: International Federation of Gynecology and Obstetrics
MMP: Matrix metalloproteinase
HCC: Hepatocellular carcinoma
